# Deregulation of MicroRNAs in Gastric Lymphomagenesis Induced in the d3Tx Mouse Model of *Helicobacter pylori* Infection

**DOI:** 10.3389/fcimb.2017.00185

**Published:** 2017-05-16

**Authors:** Pauline Floch, Caroline Capdevielle, Cathy Staedel, Julien Izotte, Elodie Sifré, Amandine M. Laur, Alban Giese, Victoria Korolik, Pierre Dubus, Francis Mégraud, Philippe Lehours

**Affiliations:** ^1^UMR1053 Bordeaux Research in Translational Oncology, Institut National de la Santé et de la Recherche Médicale, University of BordeauxBordeaux, France; ^2^ARNA Laboratory, Institut National de la Santé et de la Recherche Médicale U1212, Université de BordeauxBordeaux, France; ^3^Institute for Glycomics, Griffith UniversityGold Coast, QLD, Australia

**Keywords:** MALT lymphoma, *Helicobacter pylori*, microRNAs, apoptosis, TP53INP1

## Abstract

*Helicobacter pylori* infection is considered as an excellent model of chronic inflammation-induced tumor development. Our project focuses on gastric MALT lymphoma (GML) related to *H. pylori* infection and mediated by the chronic inflammatory process initiated by the infection. Recently, microRNAs (miRNAs) have emerged as a new class of gene regulators, which play key roles in inflammation and carcinogenesis acting as oncogenes or tumor suppressors. Their precise characterization in the development of inflammation and their contribution in regulating host cells responses to infection by *H. pylori* have been little explored. Our goal was to analyze the changes in miRNAs in a GML mouse model using BALB/c mice thymectomized at day 3 post-birth (d3Tx model) and to clarify their implication in GML pathogenesis. PCR array followed by RT-qPCR identified five miRNAs (miR-21a, miR-135b, miR-142a, miR-150, miR-155) overexpressed in the stomachs of GML-developing d3Tx mice infected by *H. pylori*. The analysis of their putative targets allowed us to identify TP53INP1, an anti-proliferative and pro-apoptotic protein, as a common target of 4 of the 5 up-regulated miRNAs. We postulate that these miRNAs may act in synergy to promote the development of GML. miR-142a was also overexpressed in mouse sera samples and therefore could serve as a diagnostic marker. *In situ* hybridization on gastric samples with miR-142a revealed a global up-regulation of this miRNA by the tumor microenvironment at the lymphoma stage. Dysregulation of miR-21a, miR-135b, miR-142a, miR-150, miR-155 could play a critical role in the pathogenesis of GML and might offer potential applications as therapeutic targets and novel biomarkers for this disease.

## Introduction

*Helicobacter pylori* is a Gram-negative bacterium that colonizes the human gastric mucosa of about 50% of the world population. The infection causes an inflammation (gastritis), which is superficial and asymptomatic at first, but may evolve toward gastric or duodenal ulcer, gastric adenocarcinoma, or gastric mucosa-associated lymphoid tissue (MALT) lymphoma (GML) (Parsonnet et al., 1991; Ferreri et al., 2009; Pereira and Medeiros, 2014).

GML is a rare consequence of a chronic inflammation of the gastric mucosa caused by *H. pylori* infection affecting approximately 0.1% of infected subjects (Kusters et al., 2006). GML is a non-Hodgkin's lymphoma characterized by infiltration and excessive proliferation of B lymphocytes in the gastric mucosa. Mechanisms underlying the initiation and progression of GML are not fully understood, but it is known that *H. pylori* infection promotes recruitment and proliferation of B cells in organized lymphoid follicles similar to intestinal Peyer's patches.

Alterations in miRNA expression have been associated with a range of cancers such as multiple myeloma (Pichiorri et al., 2008) or lung, colon, or ovarian cancer (Schickel et al., 2008). MicroRNAs (miRNAs) are small (18–24 nucleotides), non-coding RNAs that regulate the expression of target genes through translational repression and/or degradation of messenger RNA (mRNA) (Bartel, 2009). miRNAs are expressed in a tissue-specific manner and are involved in the regulation of numbers of physiological processes and pathways (Schickel et al., 2008). Aberrant miRNA expression had also been associated with lymphomas where they may function as tumor suppressor genes or oncogenes (oncomir) during human carcinogenesis (Tagawa et al., 2013; Musilova and Mraz, 2015). The association between miRNAs and cancer was first identified in chronic lymphocytic leukemia (CLL), in which the decreased levels of miR-15 and miR-16 targeting the anti-apoptotic protein Bcl2 promote lymphomagenesis (Calin et al., 2007; Zanesi et al., 2010).

Previous data have shown that several miRNAs can be differently expressed with a clear transition in miRNA expression during transition from gastritis to GML in agreement with histopathological grading (Thorns et al., 2012; Gebauer et al., 2014). In particular, miR-142a and miR-155 could potentially be involved in repressing their common target, the pro-apoptotic TP53INP1 (Tumor Protein P53 Inducible Nuclear Protein 1), thereby promoting lymphocyte proliferation (Saito et al., 2012). The upregulation of miR-142a and miR-155 in parallel with the down-regulation of miR-203, was confirmed recently by Fernandez et al., in a set of 16 GML cases compared to gastritis cases, (Fernandez et al., 2017). This last miRNA targets the oncogene ABL1 in *H. pylori* t(11;18) (q21:q21)-negative GML cases (Craig et al., 2011).

In the present study, a mouse model of lymphomagenesis described by our laboratory (Chrisment et al., 2014) was used to characterize the dysregulated miRNA at GML stage, using *H. pylori* infected BALB/c mice thymectomized at day 3 post birth (d3Tx). A large cohort of d3Tx BALB/c mice and non-thymectomized controls (NTx) were either infected with *H. pylori* or non-infected. GML-like lesions were only observed in the infected-d3Tx mice. In these animals, the gastric lamina propria was infiltrated by polymorphonuclear and lymphoid cells, the latter mainly organized in follicles composed of B cells with few infiltrating T cells. The monoclonality of infiltrating B cells was demonstrated and was strongly correlated with the presence of lymphoepithelial lesions. Using the material obtained in Chrisment et al. (2014), a set of 5 miRNAs (miR-21a, miR-135b, miR-142a, miR-150, miR-155) was identified as being over-expressed in the stomachs of the GML-developing d3Tx mice. The analysis of their putative targets allowed us to propose that these miARNs act in synergy to inhibit lymphocyte apoptosis. *In situ* hybridization using miR142a to probe gastric samples, suggests that the tumor microenvironment participates in the dysregulation of miR-142a. We also propose that this miRNA could serve as a non-invasive diagnostic marker of GML.

## Materials and methods

### Samples from mice

Experiments were performed on materials obtained from mice, as described previously (Chrisment et al., 2014). All experiments were performed in Specific Pathogen Free animal facilities at the University of Bordeaux according to EU recommendations (European Directive 2010/63/EU) on animal experimentation. This study conforms to the University of Bordeaux and the French Ministry of Agriculture Guidelines on Animal Care and the French Committee of Genetic Engineering, with respect to the principle of the 3Rs (Replacement, Reduction and Refinement; approval number 50120143-A).

### miRNA extraction

miRNAs were extracted from frozen gastric biopsies using the miRNeasy mini kit (Qiagen, Courtaboeuf, France) according to the manufacturer's instructions. miRNA samples were extracted from non-thymectomized (NTx) [7 non-infected (NI) and 19 *H. pylori* infected mice] and thymectomized mice at day 3 post-birth (d3Tx) (7 NI and 19 *H. pylori* infected mice). miRNAs were also extracted from paraffin-embedded gastric tissues using the miRNeasy FFPE kit (Qiagen). Two 20 μm sections or four 10 μm sections from 6 d3Tx mice stomachs (3 NI and 3 infected mice) were used for miRNA extraction according to the manufacturer's instructions. miRNAs were extracted from 500 μl of serum (diluted 4-fold), by adding 750 μl of TRIzol® LS Reagent (Ambion by Life Technologies, Carlsbad, CA, USA) according to the manufacturer's instructions.

### miRNA reverse-transcription

The miScript II RT kit with the HiSpec Buffer (Qiagen) was used for reverse transcription of miRNA samples prepared as described above (250 ng RNA per sample), according to the manufacturer's instructions.

### PCR array

The expression of 372 miRNAs of the gastric mucosa of infected and NI d3Tx mice was evaluated by PCR array using “Mouse miFinder 384HC miScript miRNA PCR Array” panel (MIMM-3001Z, Qiagen).

First, a PCR array with miRNAs from frozen gastric biopsies was performed. 3 NI and 4 infected d3Tx mice were included. Mice with the lowest and highest inflammatory scores in NI and infected d3Tx mice (Chrisment et al., 2014), respectively, were included. As we previously described (Varon et al., 2012; Chrisment et al., 2014), inflammation was graded on a 0–4-point scale: 0: Normal; 1: Small multifocal leukocyte accumulations in mucosa; 2: Coalescing mucosal inflammation; early submucosal extension; 3: Coalescing mucosal inflammation with prominent multifocal submucosal extension, follicle formation; 4: Severe diffuse inflammation of mucosa, submucosa, with or without involvement of deeper layers. Lymphoid infiltrates were graded on a 0–3-point scale: according to the following criteria: 0: no change; 1: single or few small aggregates of lymphocytes; 2: multiple multifocal large lymphoid aggregates or follicles; 3: extensive multifocal lymphocytic infiltration often extending through the depth of mucosa, resulting in distortion of the epithelial surface. Only RNAs of high integrity and appropriate concentration after analysis on the Agilent 2200 Tape Station (Agilent Technologies, Santa Clara, CA, USA) were included. An additional PCR array with miRNAs from paraffin-embedded gastric tissues was then performed: two miRNAs from paraffin-embedded gastric tissues obtained from infected d3Tx mice exhibiting typical GML lesions were included and compared to 2 miRNA from NI d3Tx mice.

Analysis of the expression of 372 cancer-related miRNAs and six reference miRNAs (SNORD61, SNORD95, SNORD96A, SNORD68, SNORD72, RNU6) was performed using the QuantiTect SYBR Green PCR Master Mix and miScript Universal Primer (Qiagen), according to the manufacturer's recommendations. Distribution of 0.93 ng of cDNA per well was carried out by the robot Eppendorf epMotion M5073 (Eppendorf, Hambourg, Germany). PCR arrays were performed using the CFX384™ Real-Time PCR detection system (Bio-Rad, Marnes la Coquette, France). Cycle quantification (Cq) data for each miRNAwas normalized to 4 of the most stable of the 6 reference miRNAs (variation < 0.5 Cq) and compared between NI and infected d3Tx mice according to the ΔΔCq method using the online system available for Qiagen PCR arrays users (pcrdataanalysis.sabiosciences.com/mirna/arrayanalysis.php). Values > or <3 indicated an up- or down-regulation, respectively.

### Quantitative real-time PCR

In order to confirm the results of the PCR array, the expression of miR-21a, miR-135b, miR-142a, miR-150, miR-155 in d3Tx, and NTx mice stomachs was individually performed by RT-qPCR, using the miScript Universal Primer and specific primers for each miRNA (Qiagen) at a final concentration of 0.25 μM and the SYBR® Green Premix Ex Taq™ (Tli RNaseH Plus; Takara, Saint-Germain-en-Laye, France) for qPCR of each miRNA-RT sample.

PCR reactions were carried out in duplicate in 96-well plates (Bio-Rad) with 1 ng/μl of cDNA in a total volume of 10 μl on the CFX96™ Real-Time PCR detection system (Bio-Rad) at the Quantitative PCR Platform at the University of Bordeaux (TBM-Core Real-Time PCR Platform). SNORD72 and RNU6 were used as reference genes. PCR was carried out as previously described (Floch et al., 2015).

Relative quantification of the miRNA expression was calculated for each sample, Cq-values obtained for each miRNA of interest were normalized in relation to the average of Cq-values obtained for SNORD72 and RNU6, the most stable miRNAs in gastric tissues (ΔCq = Cq_gene of interest_— Cq_SNORD72_). The 2^−ΔCq^ values were then calculated, enabling the results to be presented as relative expression levels of the miRNA of interest. Relative expression levels of each miRNA for the d3Tx mice group were also correlated with the previously obtained histological scoring (Chrisment et al., 2014). RT-qPCRs were also performed to study miRNA expression in sera samples from NI and infected d3Tx mice. Thirteen mice overexpressing at least 3 miRNAs of interest in their stomach were included and 7 NI d3Tx mice constituted the control group. The amplification conditions were the same as described above. Cq-values obtained for each miRNA of interest were normalized in relation to the average of Cq-values obtained for miR-16a (Lawrie et al., 2008).

### Bioinformatic analysis of predicted targets

An analysis of putative targets by base complementarity and conservation between species was performed for the five miRNAs studied using the Targetscan website (http://www.targetscan.org/).

### Western blotting

Gastric tissues (from 4 GML-developing infected d3Tx mice and 4 infected d3Tx mice showing no inflammation [inflammatory scores = 0) or lymphoid infiltration (lymphoid infiltration score = 0)] (Chrisment et al., 2014) were lysed in the cell lysis buffer after addition of proteinase inhibitors (Complete Mini, Roche, Basel, Switzerland), homogenized, glassbead-smashed (TissueLyser II, Qiagen) and centrifuged at 10,000 g for 10 min (4°C). The supernatants were recovered.

Protein extracts (15 μg) were separated using SDS/polyacrylamide gel electrophoresis and transferred to a nitrocellulose membrane. The membranes were incubated with the rat anti-mouse TP53INP1 monoclonal antibody (Rat F8 hybridoma culture supernatant graciously provided by Dr. Alice Carrier, CRCM, Marseille, France). Secondary Polyclonal Rabbit Anti-Rat Immunoglobulins/HRP antibody (1/1000, Dako, Copenhagen, Denmark) was used to bind primary antibody and the reaction was detected by ECL Prime Western Blotting Detection Reagent (GE Healthcare Life Sciences, Velizy-Villacoublay, France). After blotting, the membranes were stained with SYPRO Ruby Protein Blot Stain (Invitrogen) and scanned with the Molecular Imager PharosFX (Bio-Rad) for the quantification of protein load. Specific antibody signals were normalized against total protein amount for each entire lane. The signal intensities were analyzed using ImageJ software.

### *In situ* hybridization

For *in situ* hybridization (ISH) of miR-142a, deparaffinized, and rehydrated tissue sections of 6 μm thickness were digested by Proteinase K [Invitrogen, 15 μg/ml in a 5 mM Tris HCl (pH 7.4), 1 mM EDTA, and 1 mM NaCl buffer] for 20 min at 37°C, and post-fixed in 4% paraformaldehyde in PBS for 10 min at 4°C. After three washes in PBS, the tissues were prehybridized for 1 h at 55°C in 25% formamide, 4X SSC, dextran sulfate 200 mg/ml, and tRNA 220 μg/ml, and hybridized overnight at 55°C in the same buffer containing 100 nmol/l anti-miR-142a probe or 5 nmol/l anti-RNU6 probe labeled at both 3′ and 5′ ends with digoxigenin. After successive stringent washes (SSC 5X x2, SSC 1X x2, SSC 0.2X x2) at 55°C, the slides were incubated with blocking solution (ELF® 97 mRNA *In situ* Hybridization Kit, Molecular Probes, Eugene, Oregon, USA) for 1 h at room temperature. An anti-digoxigenin antibody coupled to alkaline phosphatase (Roche, Meylan, France, 1/800 dilution, overnight incubation at 4°C) was used to reveal hybridization of the anti-miRNA probes. After 3 washes, a 2 h incubation step at room temperature in 1-StepNBT/BCIP plus Suppressor Solution (Sigma-Aldrich, St-Louis, MO, USA) was performed. Slides were dehydrated and mounted with Eukit-mounting medium (O. Kindler GmbH, Freiburg, Germany).

Slides were scanned using a digital slide scanner (Panoramic SCAN; 3DHISTECH Ltd, Budapest, Hungary) equipped with a Zeiss objective (Plan-Apochromat 40; numerical aperture, 0.95; Carl Zeiss Microscopy GmbH, Jena, Germany) and a high-resolution color camera (VCC-FC60FR19CL, 4MP, CIS Corporation, Japan) available at the Experimental Histopathology Platform, US 005 UMS 3427-TBM CORE. The images were read using the Panoramic Viewer software version 1.15.4 (3DHISTECH Ltd).

### Statistical analysis

Statistical analyses were performed with GraphPad Prism 5.01 (GraphPad Software, Inc., La Jolla, CA, USA). The Mann-Whitney test was used as nonparametric test to compare the distributions of two unmatched groups. Differences were considered significant when p was inferior to 0.05 (^*^p < 0.05).

## Results

### Investigation of miRNA expression by PCR array

In order to identify the changes in miRNA expression, a PCR array with a pool of miRNAs of frozen gastric biopsies from 4 infected and 3 NI d3Tx mice was performed. Infected d3Tx mice with inflammatory scores between 3 and 4 and lymphoid infiltration scores between 2 and 3 were included and compared to NI d3Tx mice with inflammatory and lymphoid infiltration scores between 0 and 1 (Chrisment et al., 2014). Among the 372 miRNAs represented on the array, 110 were undetectable or weakly expressed (Cq-values > 30), leaving 262 miRNAs for the analysis. Among these, the expression of only 2 miRNAs (miR-135b and miR-155) was markedly increased in the infected group (Fold-regulation value >3), whereas the expression of 5 miRNAs (miR-206, miR -802, miR-122, miR-377, miR-33) was decreased (Fold-regulation value <3; Table S1).

Considering the restricted number of dysregulated miRNAs detected by the PCR array at the lymphoma stage, a second PCR array was performed using paraffin-embedded gastric tissues. miRNA expression was interrogated in the 2 of most representative of GML mice (inflammatory scores between 3 and 4 and lymphoid infiltration scores = 3). miRNA expression from a pool of RNA of 2 infected d3Tx mice was compared to a pool of RNA from 2 NI d3Tx mice (2 of the 3 mice that were included in the first PCR array run). Among the 372 miRNAs represented on the array, 136 were undetectable or weakly expressed (Cq-value > 30), leaving 236 miRNAs for the analysis. Among these, the expression of 19 miRNAs was noticeably increased at GML stage (Fold-regulation value >3), whereas the expression of 14 miRNAs was decreased (Fold-regulation value < −3; Table 1).

**Table 1:** **Deregulated miRNAs in infected d3Tx mice compared to NI d3Tx mice**.

**miRNA**	**Fold-regulation value**	
	**PCR array 1**	**PCR array 2**
miR-135b	4.655	15.614
miR-142a	1.216	12.410
miR-150	2.062	10.776
miR-19a	−1.914	8.495
miR-153	−1.915	5.541
miR-17	−1.531	5.483
miR-340	−1.208	5.095
miR-155	7.301	4.801
miR-135a	1.276	4.695
miR-19b	−2.615	4.654
miR-140	−1.482	4.595
miR-190a	−1.393	4.407
let-7c-2	−1.367	3.950
miR-101a	−2.974	3.757
miR-21a	2.415	3.735
miR-342	1.06	3.571
miR-376c	−2.209	3.252
miR-18a	−1.213	3.171
miR-126a	−1.620	3.077
let-7b	1.294	−3.064
miR-323	−1.323	−3.232
miR-125a	−1.309	−3.320
miR-615	2.212	−3.347
miR-320	1.666	−3.454
miR-375	−1.172	−3.490
miR-382	−1.759	−3.565
miR-193b	−1.182	−4.116
miR-762	−1.556	−4.502
miR-1224	−1.665	−5.071
miR-494	−2.229	−5.181
miR-802	−3.937	−5.374
miR-217	−1.824	−17.816
miR-216a	−1.093	−41.184

*PCR array 1 was performed with a pool of miRNAs from frozen gastric biopsies from 4 infected and 3 NI d3Tx mice. PCR array 2 was performed with a pool of miRNAs from paraffin-embedded gastric tissues from 2 infected and 2 NI d3Tx mice. miRNAs regulated in common between the two runs of PCR arrays are highlighted in gray*.

### Relative expression levels of miR-21a, miR-135b, miR-142a, miR150, and miR-155 in NTx and d3Tx mice

Some of the up-regulated miRNAs in GML mice identified above were investigated further using a larger number of samples in order to evaluate more precisely the level of deregulation at the lymphoma stage. These were selected on the basis of the results from both PCR arrays in correlation with the literature. The final list was: miR-21, miR-135b, and miR-155 [with fold-regulation values > 3 (Table 1)] as well as miR-142a and miR-150 as their over-expression at the lymphoma stage was described by others (Saito et al., 2012; Thorns et al., 2012; Gebauer et al., 2014; Fernandez et al., 2017).

**Figure 1:**
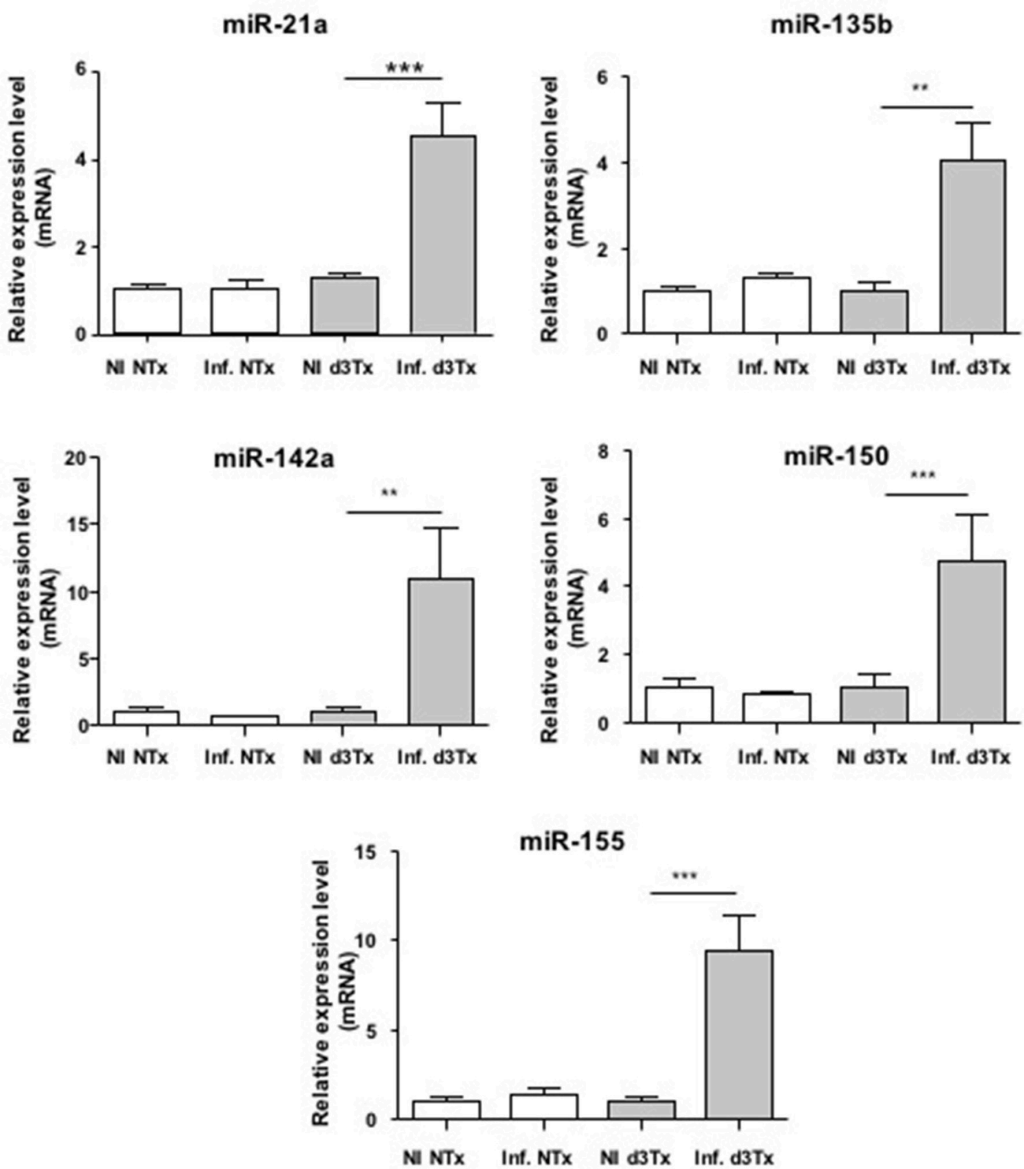
**Relative expression levels of miR-21a, miR-135b, miR-142a, miR150, and miR-155 in ***Helicobacter pylori*** infected and non-infected (NI) NTx and d3Tx mice stomachs**. Expression levels quantified by RT-qPCR for infected NTx (*n* = 19) and d3Tx (*n* = 19) mice groups were normalized in comparison to expression levels for NI NTx (*n* = 7) and NI d3Tx (*n* = 7) control groups, respectively. SNORD72 was used to normalize miRNA expression levels. The results were similar to those obtained with RNU6. Data are plotted as bar graphs displaying the mean ± standard deviation for each group, ^**^p < 0.01, ^***^p < 0.001.

Over-expression of miR-21a, miR-135b, miR-142a, miR-150, and miR-155 was confirmed at the GML stage (Figure 1) compared to d3Tx and to NTx mice (NI and infected). The relative expression levels of these miRNAs in stomachs of infected d3Tx mice were 4–11 times higher than that of NI d3Tx mice. None of these 5 miRNAs was increased in infected NTx stomachs compared to their NI counterparts.

The relative expression levels for these 5 miRNAs in infected d3Tx mice were classified according to histological scores of inflammation and lymphoid infiltrates determined previously in the laboratory (Chrisment et al., 2014). A significant increase was observed in d3Tx mice with those scores for miR-21a (Figure S1). This was not the case for the 4 other miRNAs studied (data not shown). Hence, miR-21a could be a marker of inflammation intensity during gastric lymphomagenesis induced by *H. pylori* infection.

### TP53INP1 is targeted by miRNAs over-expressed in gastric MALT lymphoma

Identification of miRNA target genes is essential for determining miRNA function. Recent studies have indicated that a single miRNA may regulate more than 200 target genes. Furthermore, one target gene could be regulated by many miRNAs. The interrogation of the TargetScan database for predicting target genes (http://www.targetscan.org) revealed that 4 of the 5 miRNAs up-regulated in our model, namely miR-135b, miR-142a, miR-150, and miR155, have potential binding sites within the 3′ UTR of the mRNA of the pro-apoptotic gene TP53INP1 and hence would be able to down-regulate it.

**Figure 2:**
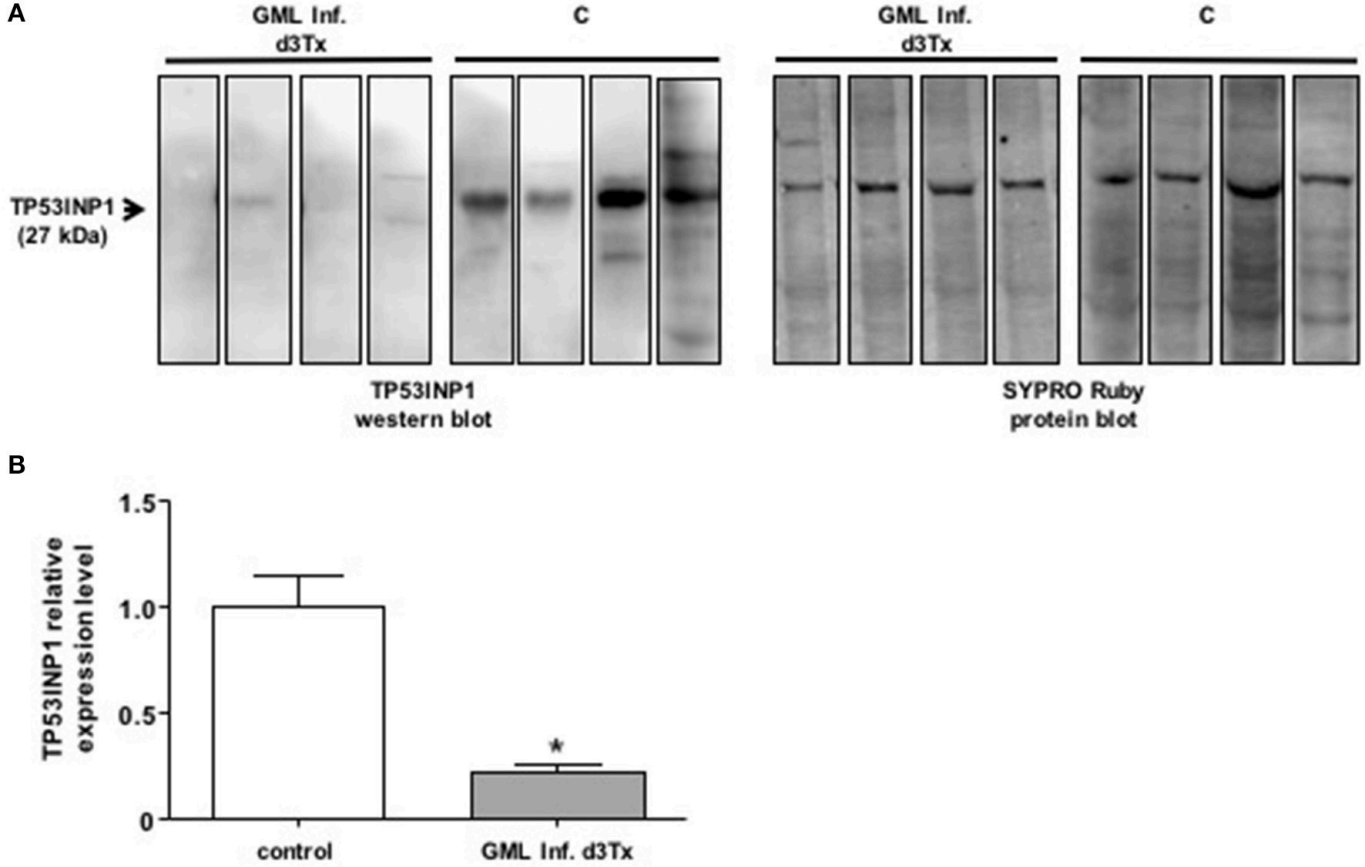
**Western blot analysis of TP3INP1 (A)** Example of Western-blot analysis of TP3INP1 in four GML-developing, *Helicobacter pylori* infected d3Tx mice compared to four infected d3Tx mice showing no gastric inflammation. Corresponding Sypro Ruby staining are shown on the side. Weak of no visible TP53INP1 was found in GML-developing mice compare to control. **(B)** Quantification of Western-blot analysis of TP3INP1 in GML-developing, *Helicobacter pylori* infected d3Tx mice (*n* = 4) compared to infected d3Tx mice (*n* = 4) showing no inflammation (inflammatory scores = 0) or lymphoid infiltration (lymphoid infiltration = 0). Intensity of Sypro Ruby staining (total protein staining) was used for normalization. The production of TP53INP1 decreased in GML-developing mice compared to that observed in control mice. Data are plotted as bar graphs displaying the mean ± standard deviation for each group, ^*^p < 0.05.

We examined the levels of production of TP53INP1 by Western blot analysis in GML-developing infected d3Tx mice. The protein levels were compared to those produced in infected d3Tx mice showing no inflammation (inflammatory scores = 0) or lymphoid infiltration (lymphoid infiltration = 0). As expected, the production of TP53INP1 was decreased in GML mice relative to control mice (Figure 2).

### miR-142a could be a diagnostic marker of gastric MALT lymphoma

**Figure 3:**
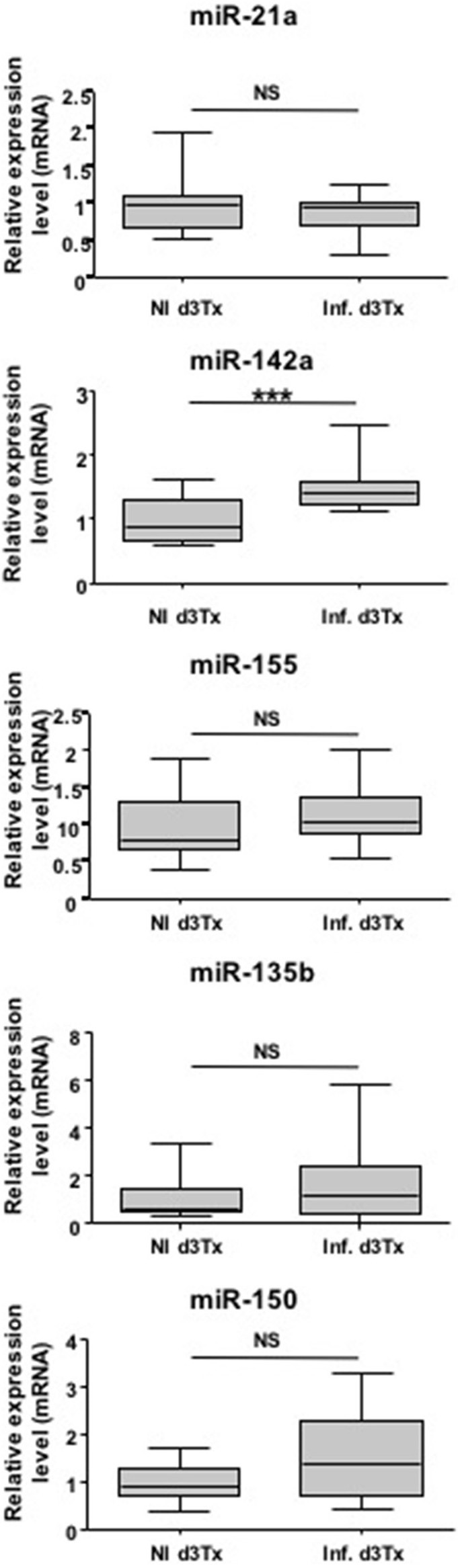
**Relative expression levels of interest miRNAs in ***Helicobacter pylori*** infected and non-infected (NI) d3Tx mice sera**. Expression levels in sera quantified by RT-qPCR for NI infected d3Tx mice group (*n* = 12) were normalized in comparison to NI d3Tx (*n* = 7) control group expression levels. miR-16a was used to normalize miRNA expression levels. Graphic representation as box plots, with the box representing 50% of values around the median (horizontal line) and the whiskers representing the minimum and maximum of all the data, ^***^p < 0.001. NS = non-significant.

miRNAs have been previously described as potential diagnostic markers of human diseases (Cho, 2010). Relative expression of miR-21a, miR-135b, miR-142a, miR-150, and miR-155 was therefore evaluated in NI and infected d3Tx mice sera by RT-qPCR. Expression of these five miRNAs was detected in mice sera, but only miR-142a expression was significantly increased in the sera of GML-developing mice compared to the control group (Figure 3).

Hence, with further confirmation, miR-142a has a potential to be a non-invasive diagnostic marker of gastric MALT lymphoma.

### miR-142a is expressed by lymphoid infiltrates and the tumor environment

In order to identify the miR-142a-producing cells, either lymphocytes or cells from the tumor microenvironment such as epithelial cells, ISH for miR-142a was performed on sections from NI and infected d3Tx mice stomachs and showed that it was, indeed expressed, by both lymphoid infiltrates and the tumor environment (Figure S2).

## Discussion

miRNAs play an important role in the regulation of the immune system (O'Connell et al., 2012) and are involved in carcinogenesis (Schickel et al., 2008). Our work focused on the dysregulation of miRNAs that could favor the emergence of GML. By using the unique material obtained from a previous study (Chrisment et al., 2014), in which we were able to induce GML in *H. pylori*-infected d3Tx mice, we showed an over-expression of miR-21a, miR-135b, miR-142a, miR-150, and miR-155 in mice stomachs at the lymphoma stage. Studying the predicted targets of these miRNAs allowed us to identify a common regulatory target, which corresponds to the TP53INP1 pro-apoptotic factor. Our work highlighted that miR-21a is a marker of intensity of gastric inflammatory scores and for the first time, indicated that miR-142a could be a non-invasive marker of GML.

**Figure 4:**
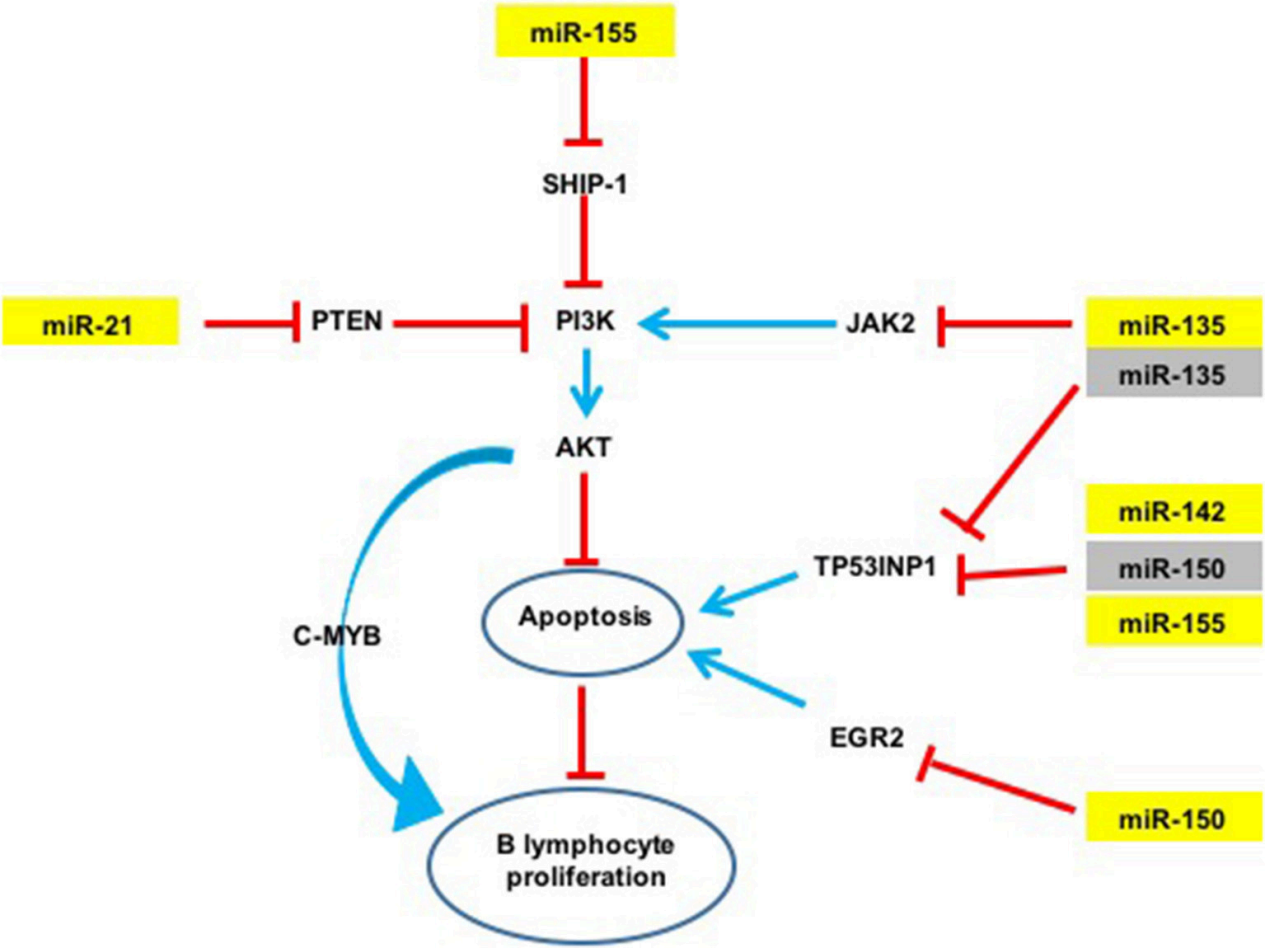
**Potential action network of predicted targets of miRNAs overexpressed in GML mice stomachs**. The five overexpressed miRNAs (in yellow) inhibit the expression of various targets such as TP53INP1. Activation of a signaling pathway is represented by blue arrows and inhibition by red arrows. Validated targets are in yellow and non-validated targets in gray.

An over-expression of miR-21a was observed in infected d3Tx mice stomachs and relative expression levels of this miRNA were correlated to histological scores of inflammation and lymphoid infiltrates determined previously in the laboratory (Chrisment et al., 2014). Expression of miR-21a increased gradually with inflammation and MALT development. miR-21a targets Phosphatase and TENsin homolog (PTEN), which specifically regulates the phosphoinositide 3-kinase (PI3K)/Akt pathway thereby inhibiting apoptosis (Sheedy, 2015) (Figure 4). This miR-21a has both pro-inflammatory and anti-inflammatory activities *in vivo*, and its dysregulation may influence homeostasis of the inflammatory response (Sheedy, 2015). In hepatocellular and gastric carcinomas, and in lymphomas such as follicular lymphomas (Zhang et al., 2008; Lawrie, 2012; Musilova and Mraz, 2015), miR-21a was shown to influence invasion, cell migration and apoptosis inhibition. Therefore, miR-21a over-expression in our GML model correlates with previously reported observations.

The expression of miR-135b is increased in various cancers such as colorectal cancers and acts as an oncomir (Valeri et al., 2014), but it has never been studied in GML. In contrast, this miRNA is also considered to be a tumor suppressor in Hodgkin lymphoma by inhibition of JAK2 expression thereby favoring apoptosis (Lawrie, 2012; Figure 4).

An over-expression of miR-150 has already been described in GML (Thorns et al., 2012; Gebauer et al., 2014), as well as in gastric carcinoma (Wu et al., 2010). miR-150 has been proposed to act as an oncomir in gastric lesions. This miRNA is considered to be a tumor suppressor gene in hematopoietic carcinoma. NK/T cell proliferation was also associated with a decrease in miR-150 expression (Watanabe et al., 2011). miR-150 down-regulates Early Growth Response 2 (EGR2) expression thereby inhibiting apoptosis (Wu et al., 2010). miR-150 was also reported to inhibit cell proliferation by targeting Myb proto-oncogene (c-Myb) (Xiao et al., 2007).

An over-expression of miR-142a and miR-155 has previously been described in GML tissues of C57BL/6 mice infected by *H. heilmanii* and in gastric biopsies of GML patients (Saito et al., 2012; Fernandez et al., 2017). miR-155 expression is increased with *H. pylori* infection in various cell types including human T-cells, primary macrophages and various epithelial cell lines, as well as in the gastric mucosa of infected mice and humans (Xiao et al., 2009; Oertli et al., 2015; Chung et al., 2017). miR-155 inhibits the NF-κB pathway by inhibiting TNF-α production (Hoces de la Guardia et al., 2013; Wang et al., 2016). It was described as an oncomir in GML (Saito et al., 2012). This miRNA can also target Src homology 2 domain–containing inositol-5-phosphatase 1 (SHIP-1) (Figure 4), which in turn inhibits the PI3K/AKT pathway (Thorns et al., 2012). It is, therefore possible, that miR-155 could inhibit apoptosis in our GML-mouse model. miR155 could also have a regulatory role in the inflammatory response by negative regulation of the release of proinflammatory cytokines and signal transduction during *H. pylori* infection (Xiao et al., 2009; Yao et al., 2015; Wang et al., 2016). *In vitro* miR-155 decreased the survival of intracellular *H. pylori* by inducing the autophagy process (Wu et al., 2016).

The activity of the miRNAs on the PI3K/Akt pathway should be considered in parallel with the activity of CagA on this pathway. Indeed, *H. pylori* manipulates this pathway through translocated CagA via phosphorylation of CagA at EPIYA-motif B (Selbach et al., 2009; Zhang et al., 2015). However, the *H. pylori* strains used in our previous study (Chrisment et al., 2014; the source of the materials interrogated in this study) are all *cag*PAI negative or harbor an inactive *cag*PAI. It is also possible that miR-150 and miR-155 could promote lymphomagenesis by acting on DNA mismatch repair pathway. These miRNAs could indeed target and modulate DNA mismatch repair genes such as POLD3 and MSH2 (Santos et al., 2017) and favor genomic instability and mutagenesis.

A single miRNA can target multiple mRNAs and one mRNA is often targeted by multiple miRNAs. The relative level of a protein levels can be considerably reduced if its corresponding mRNA is targeted by several miRNAs. miR-135b, miR-142a, miR-150, and miR155 are potentially able to bind to the 3′ UTR region of the Tumor Protein 53-Induced Nuclear Protein 1 (TP53INP1) mRNA. TP53INP1 is a protein that is over-expressed during stress responses including inflammation. It is a proapoptotic stress-induced p53 target gene. p53 activates TP53INP1 transcription, and overexpression of TP53INP1 induces cell cycle arrest and apoptosis (Tomasini et al., 2005). This target has already been validated by using Luciferase assays for miR-142a and miR-155 (Saito et al., 2012). Our results suggest that this transcript can be targeted also by two other miRNAs, i.e., miR-135b and miR-150. Expression of TP53INP1 mRNA evaluated by RT-qPCR in mice stomachs did not significantly differ between NI and infected d3Tx mice (data not shown). In contrast, TP53INP1 protein levels, evaluated by Western blot analysis, were decreased in GML-developing mice in comparison to control mice. Hence, miR-135b, miR-142a, miR-150, and miR155 seem to down-regulate TP53INP1 production via mRNA translational repression but not by mRNA degradation (Figures 2, 4). The strong inhibition of TP53INP1 production found in GML-developing mice again suggest that it could participate in the inhibition of cell apoptosis, thereby allowing acceleration of MALT lymphoma cell proliferation.

A single miRNA can act both as an oncogene (miR-21a, miR-135b, miR-142a, miR-150, and miR-155) or a tumor suppressor (miR-135b, miR-150), thus making the mechanisms involved in lymphomagenesis more complex. The miRNAs over-expressed in our study could act synergistically by targeting multiple signaling pathways (Figure 4). miR-21a, miR-135b et miR-155 indirectly target the PI3K/AKT signaling pathway: indeed, miR-21a and miR-155 activate the PI3K/AKT signaling pathway by targeting PTEN and SHIP-1 respectively, and promote cell survival (Thorns et al., 2012; Sheedy, 2015; Lu et al., 2017). On the contrary, miR-135b down-regulates JAK2 involved in the activation of this pathway (Lawrie, 2012). As previously mentioned, cell survival may also be mediated by inhibition of TP53INP1 translation by miR-135b, miR-142a, miR-150, and miR-155. Therefore, the finding that these five miRNAs are over-expressed at the GML stage in mice could represent important molecular factors in inhibition of lymphocyte apoptosis and promote B cell survival and proliferation.

Interestingly, an over-expression of miR-21a and miR-155 has also been described in NK/T cell proliferation induced by Epstein Bar Virus (EBV). These miRNAs are involved in immortalization of lymphocytes (Tagawa et al., 2013). Lymphomagenesis induced by two different pathogens with different mechanisms of action (indirect for *H. pylori* and direct for EBV) could be linked through common miRNA deregulation disrupting the same signaling pathways.

Evaluation of miRNA expression in serum samples is considered a simple and non-invasive predictive tool in the diagnosis of certain cancers or infectious diseases (Madhavan et al., 2013; Verma et al., 2016). In our study we observed that miR-142a is detectable at a significantly higher level in the serum of GML mice and thereby could be a potential biomarker of GML. This was not investigated by Saito et al. which investigated the upregulation of miR-142a in human and mice biopsies only (Saito et al., 2012). It would be interesting to evaluate miR-142a expression prior to the development of GML. From the perspective of an animal model, the presence of serum markers will allow the detection of GML development in order to better adapt the time of sacrifice. miR-142a seems to be expressed in both lymphoid infiltrates and the tumor environment and this suggests an important role of tumor microenvironment in gastric lymphomagenesis. Elevation of miR-142a in serum is an intriguing finding, not only useful in animal experiments, but which could also become useful for diagnosis of human GML. Complementary studies are indeed necessary to define sensitivity and specificity of miR-142a elevation in serum.

In conclusion, five miRNAs (miR-21a, miR-135b, miR-142a, miR-150, miR-155) are up-regulated in gastric lymphomagenesis in mice, were identified in our study. Upregulation of miR135b was never described before in GML cases and neither was the upregulation of miR-142a in mice sera. In addition, this study is in agreement with the previous findings obtained using mouse or human materials describing the upregulation of miR-142a and miR-155 at GML stage (Saito et al., 2012; Fernandez et al., 2017). Our results indicate that the miRNAs described in this study, could act synergistically on common or redundant targets and signaling pathways to promote cell survival and lymphocyte proliferation. Our findings also indicate that miRNAs deregulation may be involved in GML pathogenesis similar to that identified other cancers.

The validation of miRNA dysregulation relies on interrogation of whole stomach RNA. The problem with the current approach, is that presence of normal tissue could dilute and therefore, interfere with the identification of the changes in miRNA expression, leading to quantitative underestimation or omission of important miRNA species. Subsequent studies should be aimed at examining tissues enriched for lymphoma cells (e.g., by laser microdissection or FACS sorting). Finally, further analysis of these miRNAs in human biopsies or histological sections would validate their deregulation at the GML stage and define potential new therapeutic targets. miR-142a could also be a potential marker of GML.

## Author contributions

PF: Wrote the manuscript, performed the experiments, analyzed the data. CC: Performed the experiments, analyzed the data. CS: Wrote the manuscript, analyzed the data. JI: performed the experiments. ES: Performed the experiments. AL: Wrote the manuscript. AG: Performed the experiments. VK: Wrote the manuscript. PD: Analyzed the results. FM: Analyzed the results. PL: Designed and analyzed the experiments, wrote the manuscript.

### Conflict of interest statement

The authors declare that the research was conducted in the absence of any commercial or financial relationships that could be construed as a potential conflict of interest.

## Supplementary material

Table S1 **Deregulated miRNAs in infected d3Tx mice compared to NI d3Tx mice. PCR array was performed with a pool of miRNAs from frozen gastric biopsies from 4 infected and 3 NI d3Tx mice**.

Figure S1 **Correlation between relative expression levels of miR-21a and histological scores of *Helicobacter pylori* infected d3Tx mice**. Relative expression levels for miR-21a quantified by RT-qPCR were classified according to histological scores obtained for each infected d3Tx mouse. **(A)** Correlation of relative expression levels in comparison with inflammation scores (*n* = 8, *n* = 11 for scores of 2 and 3/4, respectively). **(B)** Correlation of relative expression levels in comparison with lymphoid infiltrate scores (*n* = 4, 10, and 5 for scores of 1, 2, and 3, respectively). Data are plotted as bar graphs displaying the mean ± standard deviation for each group, ^*^*p* < 0.05. NS = non-significant; in red, significant expression levels when compared with NI d3Tx control group (*n* = 7) (*p* < 0.05).

Figure S2 ***In situ* hybridization (ISH) of miR-142a**. Images taken by light microscopy (Nikon Eclipse 50i) with a 20X objective, and using a camera (Nikon Digital Sight DS-Fi1). The mucosa is **(A)** Example of ISH of miR-142a from a NI d3Tx mouse stomach: absence of labeling. The mucosa is oriented upwards, the muscular part downwards. **(B)** Example of ISH of miR-142a from an infected d3Tx mouse stomach: miR-142a is expressed (color brown/purple) by lymphoid infiltrates and the tumor environment. The gastric mucosa is hyperplastic compare to NI control. A star visualizes a typical Gastro Intestinal Neoplastic lesion. The lymphoid infiltrates are surrounded by a dotted line.
