# Supplementary material for: Deregulation of MicroRNAs in Gastric Lymphomagenesis Induced in the d3Tx Mouse Model of Helicobacter pylori Infection
Source: Front Cell Infect Microbiol. 2017 May 16;7:185. doi: 10.3389/fcimb.2017.00185 (PMC5432547; doi:10.3389/fcimb.2017.00185)
Supplement: Table S1 — Deregulated miRNAs in infected d3Tx mice compared to NI d3Tx mice. PCR array was performed with a pool of miRNAs from frozen gastric biopsies from 4 infected and 3 NI d3Tx mice. [file Table1.DOCX]

**Supplemental Table 1.** Deregulated miRNAs in infected d3Tx mice compared to NI d3Tx mice. PCR array was performed with a pool of miRNAs from frozen gastric biopsies from 4 infected and 3 NI d3Tx mice.

| **miRNA** | **Fold-regulation value** |
| --- | --- |
| miR-155 | 7.301 |
| miR-135b | 4.655 |
| miR-33 | -3.158 |
| miR-377 | -3.186 |
| miR-122 | -3.651 |
| miR-802 | -3.937 |
| miR-206 | -21.200 |
