# Supplementary figures and images for: Deregulation of MicroRNAs in Gastric Lymphomagenesis Induced in the d3Tx Mouse Model of Helicobacter pylori Infection
Source: Front Cell Infect Microbiol. 2017 May 16;7:185. doi: 10.3389/fcimb.2017.00185 (PMC5432547; doi:10.3389/fcimb.2017.00185)

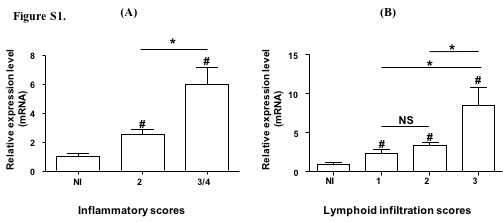

Supplement: Figure S1 — Correlation between relative expression levels of miR-21a and histological scores of Helicobacter pylori infected d3Tx mice. Relative expression levels for miR-21a quantified by RT-qPCR were classified according to histological scores obtained for each infected d3Tx mouse. (A) Correlation of relative expression levels in comparison with inflammation scores (n = 8, n = 11 for scores of 2 and 3/4, respectively). (B) Correlation of relative expression levels in comparison with lymphoid infiltrate scores (n = 4, 10, and 5 for scores of 1, 2, and 3, respectively). Data are plotted as bar graphs displaying the mean ± standard deviation for each group, *p < 0.05. NS = non-significant; in red, significant expression levels when compared with NI d3Tx control group (n = 7) (p < 0.05). [file Image1.JPEG]

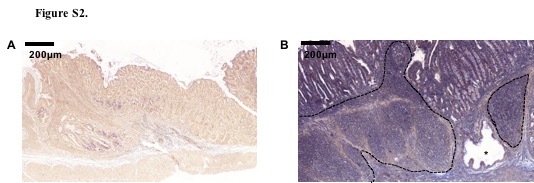

Supplement: Figure S2 — In situ hybridization (ISH) of miR-142a. Images taken by light microscopy (Nikon Eclipse 50i) with a 20X objective, and using a camera (Nikon Digital Sight DS-Fi1). The mucosa is (A) Example of ISH of miR-142a from a NI d3Tx mouse stomach: absence of labeling. The mucosa is oriented upwards, the muscular part downwards. (B) Example of ISH of miR-142a from an infected d3Tx mouse stomach: miR-142a is expressed (color brown/purple) by lymphoid infiltrates and the tumor environment. The gastric mucosa is hyperplastic compare to NI control. A star visualizes a typical Gastro Intestinal Neoplastic lesion. The lymphoid infiltrates are surrounded by a dotted line. [file Image2.JPEG]
